# Familial Periodic Fever, Aphthous Stomatitis, Pharyngitis and Adenitis Syndrome; Is It a Separate Disease?

**DOI:** 10.3389/fped.2021.800656

**Published:** 2022-03-03

**Authors:** Tamar Veres, Gil Amarilyo, Sabreen Abu Ahmad, Maryam Abu Rumi, Riva Brik, Nofar Hezkelo, Orly Ohana, Yoel Levinsky, Gabriel Chodick, Yonatan Butbul Aviel

**Affiliations:** ^1^The Ruth and Bruce Rappaport Faculty of Medicine, Technion–Israel Institute of Technology, Haifa, Israel; ^2^Pediatric Rheumatology Unit, Schneider Children's Medical Center of Israel, Petah Tikva, Israel; ^3^Sackler Faculty of Medicine, Tel Aviv University, Ramat Aviv, Israel; ^4^Department of Pediatrics B, Rambam Medical Center, Ruth Rappaport Children's Hospital, Haifa, Israel; ^5^Pediatric Rheumatology Service, Rambam Medical Center, Ruth Rappaport Children's Hospital, Haifa, Israel; ^6^Department of Medicine F-Recanati, Rabin Medical Center, Beilinson Hospital, Petah Tikva, Israel; ^7^Department of Pediatrics C, Schneider Children's Medical Center, Petah Tikva, Israel; ^8^Maccabi Institute for Research and Innovation, Maccabi Healthcare Services, Tel Aviv, Israel

**Keywords:** PFAPA, family, history, Mediterranean, fever

## Abstract

**Introduction:**

Periodic Fever, Aphthous Stomatitis, Pharyngitis, Adenitis (PFAPA) is the most common periodic fever syndrome in the pediatric population, yet its pathogenesis is unknown. PFAPA was believed to be sporadic but family clustering has been widely observed.

**Objective:**

To identify demographic and clinical differences between patients with PFAPA and a positive family history (FH+) as compared to those with no family history (FH−).

**Methods:**

In a database comprising demographic and clinical data of 273 pediatric PFAPA patients treated at two tertiary centers in Israel, 31 (14.3%) had FH+. Data from patients with FH+ were compared to data from those with FH−. Furthermore, family members (FMs) of those with FH+ were contacted via telephone for more demographic and clinical details.

**Results:**

The FH+ group as compared to the FH− group had more myalgia (56 vs. 19%, respectively, *p* = 0.001), headaches (32 vs. 2%, respectively, *p* = 0.016), and a higher carrier frequency of M694V mutation (54% vs. 25%, respectively, *p* = 0.05). Colchicine was seen to be a more beneficial treatment for the FH+ group as compared to the FH− group; however, with no statistical significance (*p* = 0.096). FMs displayed almost identical characteristics to patients in the FH+ group except for greater arthralgia during flares (64 vs. 23%, respectively, *p* = 0.008), and compared to the FH− group they had more oral aphthae (68 vs. 43%, respectively, *p* = 0.002), myalgia/arthralgia (64 vs. 19%/16%, respectively, *p* < 0.0001), and higher rates of FH of Familial Mediterranean fever (FMF) (45 vs.15%, respectively, *p* = 0.003).

**Conclusions:**

Our findings suggest that patients with a FH+ likely experience a different subset of disease with higher frequency of family history of FMF, arthralgia, myalgia, and might have a better response to colchicine compared to FH−. Colchicine prophylaxis for PFAPA should be considered in FH+.

## Introduction

PFAPA is an acronym for periodic fever, aphthous stomatitis, pharyngitis, and adenitis syndrome. Marshall et al. first described PFAPA in 1987 as a periodic fever syndrome, which is a set of autoinflammatory disorders defined by recurrent episodes of unprovoked systemic and specific organ inflammation interchanged with periods of normal health (1).

PFAPA, the most common periodic fever syndrome in children, typically presents before the age of 5 years with fevers over 38.9°C (102°F) that last from a few days to a week and recur every 3 to 8 weeks. These fevers are accompanied by either one or more of the following symptoms: aphthous ulcers—small, relatively painless lesions on the tongue and oral mucosa, pharyngitis with or without white exudates on the tonsils, and swollen cervical lymph nodes (adenitis). Between PFAPA flare-ups, children are healthy and grow and mature naturally (2).

The association between PFAPA and other periodic fever syndromes, especially FMF is well established. We have previously reported that 18.7% of patients diagnosed with PFAPA had a concomitant diagnosis of FMF (3). Another study demonstrated that in a cohort of 393 children who fulfilled the diagnostic criteria for PFAPA, approximately 20% tested positive for a mutation in either the MVK, TNFRSF1A, or MEFV genes (2).

Originally, PFAPA was believed to be a sporadic disease with no genetic heritability. However, recent cohort studies have revealed that 10 to 78% of those with PFAPA have a family member with recurrent fever (4). Manthiram et al. studied the family history in a cohort of 80 individuals with PFAPA and discovered that 18 (23%) had a family member with symptoms concurrent with PFAPA. Compared to healthy controls, these patients had a higher prevalence of parents and/or siblings with recurrent aphthous stomatitis and/or recurrent pharyngitis, as well as higher rates of siblings who had undergone tonsillectomy (5). A longitudinal study conducted by Perko et al. found that 50/64 (78%) of their cohort had a first-degree relative who had recurrent fever or underwent tonsillectomy as children (6). Di Gioa et al.'s pedigree analysis of 68 patients with PFAPA from 14 families found that the disorder displays autosomal dominant inheritance with an estimated 50% penetrance, if Mendelian genetics are assumed. In order to uncover a causative gene, Di Gioa et al. also performed whole-genome linkage analysis on seven of these families, which did not reveal a common gene mutation associated with PFAPA (7). These findings are strongly suggestive that family history and heredity are involved in the pathogenesis of this disorder, although a clear genetic cause or mutation has yet to be discovered.

The aim of our study was to identify demographic and clinical differences between patients with PFAPA who have a positive family history compared to those with no family history, to test if heritable and sporadic subtypes of this disorder exist.

## Materials

Retrospective data was collected from 273 children diagnosed with PFAPA who were treated at one of two pediatric rheumatology clinics in Israel, the Ruth Rappaport Children's Hospital at Rambam Health Care Campus in Haifa or Schneider Children's Medical Center of Israel in Petah Tikvah, during March 2014–March 2019. Inclusion criteria for the study were clinical diagnosis of PFAPA by a pediatric rheumatologist and absence of other autoinflammatory manifestations.

All tracked cases of PFAPA were validated according to previously established clinical criteria (8).

### Methods

Information regarding demography, ethnicity, diagnosis, follow-up, treatment, genetics, and family history of PFAPA, FMF, and tonsillectomy was collected at patient clinical visits. Fifty three patients with a concurrent diagnosis of Familial Mediterranean fever (FMF), according to the pediatric Yalcinkaya-Ozen criteria or Tel Hashomer (9) criteria for FMF were excluded from this study.

**Figure 1:**
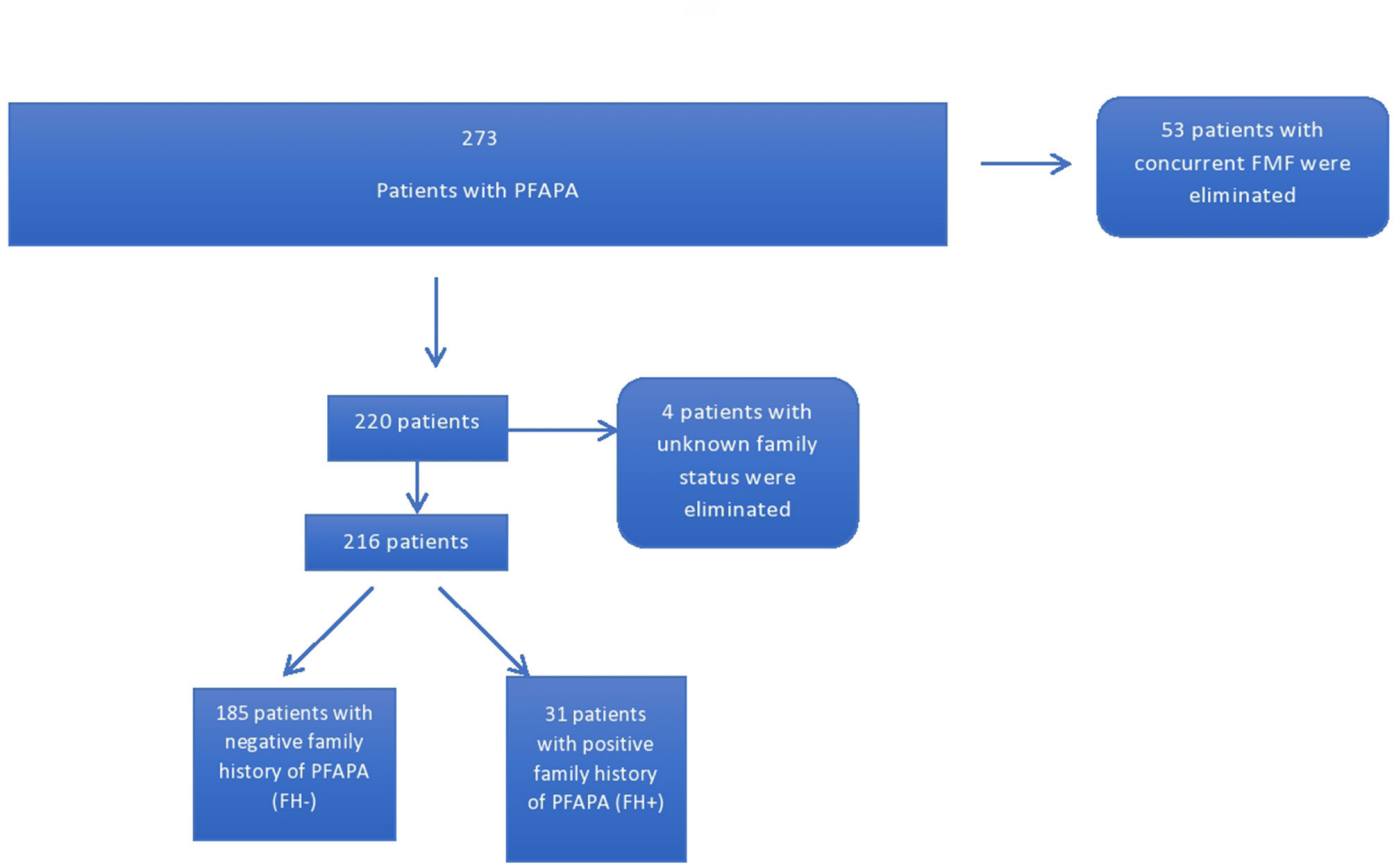
Patients with family history of PFAPA from cohort of patients with PFAPA.

Patients were stratified into two groups based on the presence of at least one relative (first or second degree) with a diagnosis of PFAPA. One hundred and eighty five patients had no family members with PFAPA while 31 had a positive family history. Four patients were eliminated due to unknown family status (Figure 1). Furthermore, the parents of patients identified as having FH+ for PFAPA were contacted and sent questionnaires inquiring about demography, clinical experience with PFAPA, and relation to the index case of the other family member with PFAPA. Eight parents could not be contacted or declined to participate in the study. A total of 23 questionnaires were filled out by either the parent or the family member depending on age and ability. The family members included 14 siblings, one parent, eight first cousins (one patient had two cousins with PFAPA), and one aunt of the original index cases.

### Statistical Analysis

Statistical analysis and comparison were performed between the FH− group and the FH+ group, as well as for the FH− and FH+ in the family members (FMs) group. Data was statistically analyzed using the SPSS statistical package, version (SPSS Inc., Chicago, Illinois). All data are expressed as median, mean ± standard deviation, or percentages. A χ2 test, Mann-Whitney U test, and Student *t*-test were used; *P* < 0.05 was accepted as statistically significant.

Each hospital's local Helsinki Committee (REB no. 0318-16 RMB and 104-16-RMC) approved the study.

## Results

The 216 PFAPA patients were divided into two groups: the FH− group (*N* = 185), where patients did not have any family members with a known diagnosis of PFAPA, and the FH+ group (*N* = 31) where each patient had at least one family member with a known diagnosis of PFAPA. Two members of the FH− group were homozygous for a mutation in one of their MEFV genes (M694V and V726A genes), however their symptomology was congruent with a diagnosis of PFAPA and not FMF as per diagnostic criteria.

Clinical and demographic characteristics of the FH+ and FH− groups are presented in Table 1. Patients in the two groups had a similar average age of symptom onset. In both groups, approximately 1.5 years elapsed from symptom onset until a diagnosis of PFAPA was conferred. Without treatment, members of both groups experienced acute attacks that lasted on average for 4 days with maximum fevers reaching 39.7C ± 0.55 in the FH− group and 39.9C ± 0.78 in the FH+ group (*P* = 0.20).

**Table 1:** Demographic characteristics of FH− and FH+ groups.

**Characteristic, *N* (%)**	**Group 1; FH−(*N* = 185)**	**Group 2; FH+ (*N* = 31)**	***P*-value**
Gender			0.32
Male	111 (60)	22 (71%)	
Female	74 (40)	9 (29%)	
Age first symptoms (years)	3 (1.5 ± 4.4)	2.8 (1.5 ±−4.1)	0.43
Age at diagnosis (years)	4.58 (3 ± 6.2)	4.75 (3 ± 6.8)	0.95
Duration of episode in days–median (max-min)	4 [3–4.5]	4 [3–7]	0.77
Interval between episode in weeks at presentation median (max-min)	4 [2.5–4]	3 [2–4]	0.27
Consanguinity	8 (4)	1 (3)	0.81
Origin, *N*	179	30	
Ashkenazi Jewish	13 (7)	3 (10)	0.71
Sephardic Jewish	66 (37)	15 (50)	0.22
Mix Sephardic and Ashkenazi Jewish	57 (32)	9 (30)	1.00
Arab	43 (24)	3 (10)	0.099

Table 2 lists the symptoms experienced by patients in both groups during PFAPA flare-ups. Headache and myalgia were experienced by a statistically greater percentage of patients in the FH+ group compared to in the FH− group (*P* = 0.0016 and *P* = 0.001, respectively).

**Table 2:** Symptoms experienced during episodes.

**Characteristic, *N* (%)**	**Group 1; FH−(*N* = 185)**	**Group 2; FH+ (*N* = 31)**	***P*-value**
Pharyngitis	170 (92)	29 (100)	0.23
Adenitis	88 (48)	19 (65.5)	0.11
Aphthous stomatitis	62 (33.5)	12 (43)	0.39
Abdominal pain	91 (49)	11 (41)	0.54
Headache	23 (12)	8 (32)	0.016
Myalgia	35 (19)	15 (56)	0.001
Arthralgia	29 (16)	6 (23)	0.39
Rash	11 (6)	2 (8)	0.66

The vast majority of patients in both groups were treated with steroids that contributed to positive clinical outcomes such as suspending or shortening the PFAPA flare-up, although increased frequency of flares occurred in 45% of patients in the FH− group and 33% in the FH+ group (*P* = 0.35) as a result of steroid usage. Colchicine was seen to be a more beneficial treatment in the FH+ group as opposed to the FH− group, although statistical significance was not reached (P=0.096). Data on response to therapy is presented in Table 3.

**Table 3:** Treatment and response among PFAPA patients with and without family history of PFAPA.

**Characteristic, *N* (%)**	**Group 1; -FH (*N* = 185)**	**Group 2; +FH (*N* = 31)**	***P*-value**
Treatment with steroids	169 (96)	28 (97)	1.00
Treatment with cimetidine	8 (4)	1 (3)	1.00
Treatment with colchicine	46 (27)	11 (37)	0.28
Treatment with montelukast	6 (3)	4 (13)	0.039
Treatment with tonsillectomy (with/without adenoidectomy)	11 (6)	1 (3)	1.00
Episode resolves with use of steroids	133 (87)	22 (92)	0.74
Episode shortened with use of steroids	149 (99)	22 (96)	0.25
Increased episode frequency with steroids	63 (45)	7 (33)	0.35
Episode frequency after steroids (in weeks), mean[range]	2.5 [1.5–4]	2.0 [2–3]	0.90
Episode stop/less frequent with cimetidine	2/8 (25)	0	1.00
Episodes stop/less frequent with colchicine	24/46 (52)	9 /11(82)	0.096
Episode stop/less frequent with montelukast	3/6 (50)	2/4 (50)	1.00
Episodes cease with tonsillectomy (with/without adenoidectomy)	3 (21.4)	0	1.00

Genetic testing for known mutations in FMF-causing genes was conducted in 34% of those in the FH− group and 41.9% in the FH+ group. Of those tested, 49% of FH− and 77% of FH+ patients were found to contain at least one such mutation (*P* = 0.12). The M694V heterozygote mutation was the predominant mutation in both groups, representing 25 and 54% of mutations in the FH− and FH+ groups, respectively (*P* = 0.053). Family history of FMF was recorded in 30% of those in the FH+ as opposed to 15% in the FH− group (*P* = 0.096).

Family history of tonsillectomy/adenoidectomy was similar in both groups with 34% in the FH− group and 15% in the FH+ group (*P* = 0.33) reporting a family member who underwent one of these procedures.

Demographic, clinical, and family history data collected from family members are displayed in Supplementary Table 1.

Family members were found to display almost identical clinical characteristics as patients in the FH+ group, except for arthralgia, which manifested in 64% of family members as compared to 23% of FH+ (*P* = 0.008). Compared to the FH− group, family members experienced more oral aphthae (68 vs. 43%, respectively, *p* = 0.002), myalgia (64 vs. 19%, respectively, *p* < 0.0001), and arthralgia (64 vs. 16%, respectively, *p* < 0.0001), as well as a higher rate of family history of FMF (45 vs. 15%, respectively, *p* = 0.003).

## Discussion

Inherited periodic fever syndromes are understood to be caused by a monogenic gene mutation which leads to innate immunity dysfunction. Homozygous carriers of such mutations have aberrant activation of their inflammasome that triggers dysregulated activation and release of pro-inflammatory cytokines, such as IL-1β that lead to symptoms of system-wide inflammation (10). PFAPA possesses clinical similarities to these monogenic periodic fever syndromes and PFAPA attacks have been shown to resolve with inhibition of IL-1 (11). However, unlike in the hereditary periodic fever syndromes, no single gene mutation has been implicated as the culprit of PFAPA and its mode of heredity is unknown (7).

Many PFAPA patients have been found to be heterozygous carriers of gene mutations responsible for these various recessively inherited periodic fever syndromes. A study in Switzerland found an increase in the expected carrier rate of NLRP3 inflammasome gene mutations, which are responsible for the majority of cases of cryopyrin-associated periodic syndrome (CAPS) in PFAPA patients compared to the general population (11). In a recent study, Manthiram et al. found genetic similarity between PFAPA and other disorders that cause recurrent inflammation in the oropharyngeal mucosa, namely aphthous stomatitis and Behçet's disease (12).

In this database, as previously presented, 18.9% of the PFAPA patients had a co-diagnosis of FMF.

In our cohort, patients were tested for common MEFV mutations that are responsible for the development of FMF. In Israel, the carrier rate of FMF-associated genes is as high as one in three in Jews of Iraqi descent (13). Different point mutations in the MEFV gene are observed in individuals of different ethnicities and all lead to the same classic disease phenotype. The carrier rate found in this cohort (of those tested for MEFV mutations) in both the FH− and FH+ groups was found to be higher than one in three with an even higher prevalence in the FH+ group compared to the FH− group, although significance was not reached. In Israel, PFAPA is found to be most prevalent in Jews of Sephardic descent (3); however, this is not enough to explain the extremely high carrier rates observed in our cohort. Furthermore, studies have found that those with a family history of PFAPA are more likely to be carriers of genes implicated in periodic fever syndromes (11).

The association between high carrier rates of MEFV mutations in those with PFAPA has been previously stated as in an early study conducted by Padeh et al. In this report, the genome of 28 PFAPA patients of Arab and Jewish origin were analyzed for the presence of one of the three most common MEFV mutations. Six (21%) were found to be heterozygotes for M694V and it was claimed that this carrier rate might be the same as the rate in the normal population and that the association required further analysis (14).

Another study by Haytoglu et al. found 34.3% of MEFV gene variants in a group of PFAPA patients, which is higher than the reported 20% carrier rate in the healthy population (15).

This association was also reported in a small Japanese cohort where the frequency of E148Q-L110P was observed in 35% of PFAPA patients, a percentage that is significantly higher than the 13% observed in healthy subjects (16).

It has been hypothesized that heterozygote carriers of PFS gene mutations are in a constant “pro-inflammatory” state and thus are susceptible to triggers that lead to symptoms of system-wide inflammation and the development of PFAPA to which a non-carrier would be immune (11).

The presence of PFS gene mutations has been shown to impact PFAPA's clinical presentation as well. FMF gene mutations, especially the M694V substitution (most prevalent in our cohort and associated with the most severe phenotype of FMF) has been observed to play a protective role in PFAPA symptomology, such as shorter PFAPA flares, decreased presence of oral aphthae, and a positive response to lower corticosteroid dosage. Some researchers believe that the pro-inflammatory state that carriers of PFS genes find themselves in actually raises the threshold for the triggering of autoinflammatory attacks and therefore these patients experience fewer symptoms (17).

Other studies indicate that FMF gene mutation carriers have earlier PFAPA disease onset and are more likely to experience symptoms associated with FMF such as abdominal pain, rash and arthralgia during PFAPA flares as compared to non-carriers (2).

In our cohort, those with FH+ experienced greater rates of myalgia and headache and exhibited better response rates to colchicine as compared to the FH− group. All other clinical characteristics between the FH+ and FH− were found to be similar or did not reach statistical significance.

A recently published report evaluating the predicting factors for resolution of PFAPA within 4 years of disease onset in a cohort of 466 Turkish PFAPA patients found that age at disease onset, positive family history of PFAPA, myalgia, and headache were all found to be related to early resolution of PFAPA syndrome (18). These findings support our hypothesis that those with PFAPA who have a positive family history of the disorder may comprise a separate group from those with PFAPA with no reported family history of the disorder and may more commonly report myalgia and headache during PFAPA flares. In our cohort, we did not complete long term follow up of the patients and thus are unable to report the effect of family history of PFAPA on the duration of the disease.

In a study performed by Butbul et al. that used the same database as in this study, it was found that patients with concurrent clinical FMF and PFAPA experienced myalgia during PFAPA attacks more frequently than their counterparts with a sole diagnosis of PFAPA (3). Although patients with clinical FMF were excluded from this study, the large presence of those with MEFV gene mutations in the FH+ group can perhaps explain this phenomenon. Colchicine, the treatment of choice for prophylaxis of FMF flares and prevention of amyloidosis has been shown to be a more effective treatment for PFAPA flare prophylaxis in those with FMF gene mutations (19); this may partially explain the differences in the response to colchicine therapy between the FH− and FH+ groups.

Data from the family members of the FH+ group was collected in the hope of providing further evidence to the hypothesis that families with multiple PFAPA sufferers experience a specific genetic subtype of the disorder that differs from a non-heritable subtype. Shortcomings of the family member group include the mode of data collection and lack of genetic testing. Parents/guardians answered a telephone questionnaire about the child's PFAPA experience without the patient ever being examined by one of the physicians involved in this study. Presence of recall and information biases has been shown to be present in parents with more than one child with the same disease. Repeat contact with the health care system instills symptom awareness and knowledge that is usually absent when a family is experiencing a disease for the first time; this can lead to over reporting of symptoms. FMs were not genetically tested for MEFV mutations (or for mutations in other PFS-causing genes) and it is not known whether family members contain these mutations in the same proportion as their relatives in the FH+ group. This information could aid in further elucidating whether a family history of PFAPA is associated with genetic mutations in FMF or other periodic fevers genes.

Demographic, clinical, and genetic data of patients in the FH+ and FH− groups does demonstrate differences, namely increased rates of myalgia and headache during flares, increased rates of M694V mutations, and a trend toward increased response rates to colchicine in the FH+ group as compared to the FH− group. When further comparing family members of the FH+ group to the FH+ group itself, the only difference discovered was that of increased arthralgia in the FM group. When comparing the FMs to the FH− group, increased rates of oral aphthae, arthralgia, myalgia, and family history of FMF were observed.

Taken together, the data does indicate that those with positive family history for PFAPA demonstrate some clinical differences from those with no family history. However, there is not enough evidence to clearly state that these two groups experience a different subset of PFAPA.

The higher rates of M694V FMF mutations in those with a positive family history should be explored further and genetic testing for family members should be completed, to derive a clearer picture of this association. Prescribing colchicine as a prophylaxis for PFAPA attacks should be considered in those with a family history of the disease.

## Data Availability Statement

The raw data supporting the conclusions of this article will be made available by the authors, without undue reservation.

## Ethics Statement

The studies involving human participants were reviewed and approved by Rambam Ethics Committee REB no. 0318-16 RMB and Schneider Children's Medical Center of Israel 104-16-RMC. Written informed consent to participate in this study was provided by the participants' legal guardian/next of kin.

## Author Contributions

TV wrote the first draft of the work. YB and GA designed and conducted the study and collected the data. All other authors helped with collecting and analyzing the data. All authors agreed to be accountable for the content of the work.

## Conflict of Interest

The authors declare that the research was conducted in the absence of any commercial or financial relationships that could be construed as a potential conflict of interest.

## Publisher's Note

All claims expressed in this article are solely those of the authors and do not necessarily represent those of their affiliated organizations, or those of the publisher, the editors and the reviewers. Any product that may be evaluated in this article, or claim that may be made by its manufacturer, is not guaranteed or endorsed by the publisher.
