# Supplementary material for: Familial Periodic Fever, Aphthous Stomatitis, Pharyngitis and Adenitis Syndrome; Is It a Separate Disease?
Source: Front Pediatr. 2022 Mar 3;9:800656. doi: 10.3389/fped.2021.800656 (PMC8929572; doi:10.3389/fped.2021.800656)
Supplement: Supplementary file 1 [file Table_1.docx]

Supp. Table 1: Family member data

|  | Family members; N=24 |
| --- | --- |
| Gender | Male 19 (79%) |
| Consanguinity | 0 |
| Origin  Ashkenazi Jewish  Sephardic Jewish  Mix Ashkenazi/Sephardic Jewish  Arab | N=23  3 (13%)  12 (52%)  4 (17%)  4 (17%) |
| Age at presentation of first symptoms | 3 [2-4.5] |
| Age at diagnosis | 4 [3-5.5] |
| Duration of episode in days | 4 [3-6] |
| Interval between episode in weeks at  presentation | 4 [3-4] |
| Pharyngitis | 20 (91%) |
| Adenitis | 14 (64%) |
| Aphthous stomatitis | 15 (68%) |
| Abdominal pain | 15 (68%) |
| Headache | 0 |
| Myalgia | 14 (64%) |
| Arthralgia | 14 (64%) |
| Rash | 1 (5%) |
| Family history of FMF | 9 (45%) |
